# Feeling the fear of many: orienting affects in Swedish austerity politics

**DOI:** 10.3389/fsoc.2025.1411526

**Published:** 2025-05-22

**Authors:** Christine Bylund

**Affiliations:** Department of Historical, Philosophical and Religious Studies, Umeå University, Umeå, Sweden

**Keywords:** affect, austerity, welfare state, disability, orientation, crip phenomenology, Sweden

## Abstract

This article investigates the emotional consequences of austerity politics targeting services and support for disabled citizens in Sweden, contributing to ongoing debates in disability studies and welfare state governance. Drawing on theories of crip phenomenology, the study focuses on how austerity policies produce affective responses—particularly fear—among disabled individuals. Based on qualitative interviews, the empirical material was collected from disabled citizens navigating the Swedish welfare system under intensified austerity measures. The research examines how these citizens experience the impact of policy reforms and the bureaucratic implementation of support reduction. The results reveal a pervasive sense of fear, disorientation, and existential insecurity, as well as increased instances of bodily harm. These affects are linked to the experience of bureaucratic violence and ableist discourse embedded in the governance of welfare services. Participants describe how these dynamics constrain their capacity to imagine and pursue viable personal futures. The article argues that austerity-driven policy changes have reshaped not only the material conditions of disabled citizens but also their emotional and social lives. It challenges the notion of ‘Swedish exceptionalism’ by illustrating how bureaucratic violence disrupts disabled individuals’ experience of full citizenship. These findings offer new insight into the relationship between affect, power, and policy in a contemporary welfare state context.

## 1 Introduction

The past two decades of global austerity have rekindled an academic interest in the relationship between the welfare state as a political and bureaucratic mechanism of economic stratification and the practical and emotional conditions it produces in citizens’ everyday lives. This leads us to ask: *How does austerity make a person feel?* Previous research has shown that austerity measures that target services and support for disabled citizens produce a range of emotions, including dread, shame, fear, grief, and anger in disabled individuals (McRuer, 2018; Ryan, 2019; Norberg, 2019). It has also demonstrated that austerity reproduces hegemonic discourses that position disabled citizens as ‘counterfeit citizens’, ‘burdens’, and ‘parasites’ (Hughes, 2015; McRuer, 2018).

Saffer et al. (2018) have shown how feelings of fear and anxiety were central to the experience of disabled citizens who found themselves in need of services and support from the welfare state of the 2010s United Kingdom. Among the range of emotions elicited by austerity politics, *fear* emerges as particularly significant due to its ability to reflect both individual vulnerability and systemic precarity. As Berezin (2002, 37) claims, it is apparent that “some emotions are more relevant to politics than others” and that “some emotions are more likely than others to emerge in the political sphere and have discernible political consequences.”

In the material analyzed for this article —consisting of interviews with disabled citizens in Sweden in need of state support and services —fear was the predominant emotion. The interviewees expressed fear in relation to previous experiences of applying for or reassessing support and service, as well as fear for present changes to eligibility criteria and for future changes to the welfare state’s support. By focusing on fear, the present study highlights its dual role (i) as an affective response to austerity measures and (ii) as a political tool that shapes and informs disabled citizens’ everyday lives and social position. In the interviews, fear not only emerges as a central emotional response but also configures the orientation of disabled citizens, thus determining how they navigate and relate to the welfare state, society, and their personal futures.

The need for studies of affect and disability becomes evident when one examines the effects of austerity politics on disabled citizens. For example, Goodley et al. (2018) argue that affect theory and the study of emotions should be a central component of disability studies since affect is crucial to the stigmatization that forms around disabled bodies. Consequently, studies of affect are not merely investigations into individual psycho-emotional reactions but constitute analyses of how these emotions are produced, how they correspond to economic and cultural structures, and how they are distributed across society.

### 1.1 Aim

This study contributes to and develops research on affect and disability in sociology by analyzing accounts of fear provided by disabled citizens who require welfare state support in Sweden. Drawing on material collected between 2017 and 2019, and focusing on fear as an affect produced by austerity politics—as well as a political emotion with both individual and collective consequences—this study investigates how austerity measures shape and inform the emotional experiences of disabled citizens.

The central questions addressed by this study are:

- How is fear expressed by disabled citizens who are affected by austerity measures?
- How does fear impact the lives of the interviewees?
- What orientations does this fear produce?

### 1.2 Background: disability and the changing Swedish welfare state

The Swedish welfare state has been of interest to sociologists for several reasons. Despite, having been characterized as a well-functioning system of stratification (Esping-Andersen, 1996), the Swedish welfare state’s standing has also been subject to debate in research that has examined how the early Swedish welfare state was built on a program of highly repressive social engineering influenced by eugenics, race-biology, and social Darwinist motives (Lucassen, 2010; Norberg, 2019).

During the early years of the Swedish welfare state, services and support for disabled citizens were primarily concerned with the provision of pensions for those who acquired their disability whilst working, i.e., a form of worker’s compensation. From the 1940s and 1950s, a large-scale institutionalization of (predominantly) disabled children took place. In these state institutions, education and health care were provided, but the surrounding society remained, in the main, inaccessible. Following the introduction of the ‘principle of normalcy’ (Lewin, 2021; Bylund, 2022) in the late 1960s, arguing that the institutionalization of disabled citizens was immoral, and the Marxist disability rights movement ‘Anti-Handikapp’, who established that the marginalization that disabled citizens faces is shaped by an inaccessible society, many of the institutions for disabled children and adults were dismantled. In the late 1970s and early 1980s several support systems were implemented, including residential care arrangements outside the state’s large-scale institutions. However, support that would grant disabled citizens self-determination was not implemented (cf. Bylund, 2022).

In the late 1980s, mobilization by Swedish disability rights movements resulted in legislation that regulated the provision of support for disabled citizens, often understood as enjoying a peak in 1994 with the implementation of the LSS Act [the Law Regulating Support and Service to Persons with Certain Functional Disabilities]. LSS grants support such as personal assistance and guidance services for disabled citizens with the goal of independent living and inclusion in society (Bylund, 2022; Norberg, 2019; Hultman, 2018). Implementing the LSS Act marked a shift in Swedish disability politics, centering around a social model of disability and framing services and support for disabled citizens as a question of democratic and civil rights. Services and support for disabled citizens were understood as being in line with the provision of a general safety net for citizens provided by a welfare state.

However, the reforms mentioned above faced opposition during their initial years of implementation, as some considered them too costly for the welfare state economy. As a result, in 1996, eligibility for personal assistance under the LSS Act was redefined based on the concept of ‘basic needs’ (Bylund, 2022; Lewin, 2021). Alongside this definition, a division was introduced between those requiring more than 20 h per week of assistance with basic needs and those requiring less. If a person’s needs exceeded this threshold, support was to be funded by the state through the Swedish Social Insurance Agency, Försäkringskassan. If the needs fell below the threshold, the municipality where the person resided was responsible for providing support.

From the late 2000s and onwards, following global austerity measures, a shift towards a neo-liberal focus has taken place that has entailed easing citizens’ tax burden (Norberg, 2019; Bylund, 2022). Consequently, debates on the cost of services and support for disabled citizens have resurfaced. Austerity measures, from 2009 and onwards, have been aimed directly at reducing services and support for disabled and chronically ill citizens. These measures have primarily focused on changing the eligibility criteria for sick benefits provided by the social insurance agency Försäkringskassan, and services and support mandated by the LSS Act (Norberg, 2019; Altermark, 2020; Lewin, 2021). Norberg (2019) has shown that politicians do not explicitly announce Swedish austerity measures to the Swedish population. Instead, they result from political pressure that is exerted on various authorities. Austerity measures have been implemented through bureaucratic and legal arrangements, for example, in changes in legal praxis and in the bureaucratic definition of ‘basic needs’ in the LSS Act (Berggren et al., 2021). A key aspect of these changes has been the 20-h-per-week threshold for basic needs in order to obtain personal assistance from the state, which has played a crucial role in the implementation of austerity policies. The shift in eligibility criteria has progressively narrowed the definition of basic needs—for instance, dressing no longer includes putting on coats or shoes, and eating excludes plating or cutting food. As a result, many individuals who previously qualified for state-funded support through Försäkringskassan have been excluded. These changes have led to thousands of disabled citizens either losing their support and services entirely or facing substantial reductions in the support they receive (Norberg, 2019; Berggren et al., 2021; Lewin, 2021). Furthermore, by the state’s use of invasive tools for the assessment and re-assessment of a person’s needs that breach their personal integrity, disabled citizens who require support and services are not only put in precarious living conditions but also find themselves under immense emotional pressure. In this article, the term ‘contemporary austerity’ refers to the ongoing political and bureaucratic transformations that were initiated in 2009 and continue to shape policy and practice in the present.

Norberg (2021, 662–664) has labeled the bureaucratic implementation of these austerity measures as ‘bureaucratic violence’. Norberg (2021, 656) states that “[s]ociological attention to bureaucratic violence is important as the technocratic veneer of bureaucracy obscures the structural and material violence enacted and contributes to its mundane appearance.” Following Norberg, I claim that more research should be conducted in this area, especially on the emotions and affects that are produced by Swedish austerity, if we are to fully understand the violence enacted on disabled citizens through austerity measures.

### 1.3 Disposition

The following section presents the theoretical framework of the study. This is followed by a description of the data collection methodology and the data analysis. The study’s findings are organized around four central themes, namely: (i) Traded narratives, (ii) Objects of fear, (iii) Wounding affects, and (iv) Disorienting affects. Each of these themes elucidates different aspects of the emotional landscape experienced by disabled citizens amidst the prevailing austerity politics that inform public and private life in Sweden. The study concludes with a discussion section that contextualizes the results within the existing literature on the topic and proposes avenues for future research into the complex dynamics that exist between power, discourse, and emotions in contemporary welfare states.

## 2 Theoretical framework

The following section outlines the theoretical perspectives that inform the analysis, focusing on the key concepts of ‘ableism’, ‘affect’, and ‘combat breathing’.

### 2.1 Ableism as a hegemonic discourse

Discourse is the key mechanism through which power operates within society, shaping our understanding of reality via language, actions, and representation. Discourse not only reflects existing power relations but also reinforces them by constructing oppositional ‘others’ and influencing how individuals perceive themselves and others (Foucault, 2010). Foucault argues that hegemonic discourse materializes in *biopolitics*—i.e., the regulation of populations and bodies by state institutions—as a central feature of modern governance. Biopolitical mechanisms, such as disciplinary practices and technologies of surveillance, operate through discourse to govern and control populations. In this way, discourse and biopolitics constitute integral components of modern power relations, shaping individual subjectivities and broader socio-political structures (Foucault and Senellart, 2010).

‘Ableism’, as developed by McRuer (2006, 2018), Campbell (2009), and Kafer (2013), can be understood as a hegemonic discourse that forms a system of discrimination and prejudice by privileging able-bodied individuals while marginalizing and oppressing those understood as ‘disabled’. Ableism is deeply ingrained in societal structures, norms, and attitudes, perpetuating the notion that able-bodiedness is inherently superior and desirable. It manifests in various forms—including physical barriers to access, unequal opportunities for employment and education, and harmful stereotypes that perpetuate stigma and exclusion. McRuer (2018) has shown how ableist discourse underpins neoliberal austerity politics by promoting and safeguarding able-bodied citizens’ safety and desires. Similarly, Goodley et al. (2018) have explored how ableist discourse interacts with the neoliberal welfare state’s emphasis on autonomy, self-sufficiency, and independence. As Goodley et al. (2018, 210) argue, this discourse fosters “the elision of individual and national economic independence with an individual and cultural celebration of autonomy.” Although Norberg (2019) applies the term *disablism* to refer to the stigmatizing discourse aimed at disabled people, while *ableism* promotes the hegemony of able-bodiedness, she makes similar claims regarding the idea that the stigmatization of disabled people is a driving force in neoliberal austerity. This stigmatization is produced and based on affect, an observation discussed in the following section.

### 2.2 Affect

Seyfert (2012, 32) describes an *affect* as something that “defines and ceaselessly constitutes and reconstitutes the nature of a body.” Furthermore, distinctions are sometimes drawn between *emotion as a sociological expression of feeling* and *affect as a biological response* (Gorton, 2007). However, regardless of one’s perspective, affect is always entangled with discourse, power, and the production of emotive states. In this vein, Gorton (2007, 334) notes that “feeling is negotiated in the public sphere and experienced through the body.” Similarly, Pedwell and Whitehead (2012, 116) argue that “power circulates through feeling” and that “politically salient ways of being and knowing are produced through affective relations and discourses.”

The present study employs Sara Ahmed’s theorization of the relationships between discourse, affect, and orientation. According to Ahmed (2014), affects are not merely expressions of subjective experience; they emerge from and reproduce power structures. Consequently, affects are deeply intertwined with discourse and materialize as emotional states, both physically and existentially. Ahmed further observes that “[e]motions[…]involve bodily processes of affecting and being affected” (Ahmed, 2014, 208), indicating that affect circulates between the subject and discourse.

Ahmed (2004a) also posits that affects are productive in the sense that they orient different (types of) bodies toward or away from specific places and spaces. In *Orientations: Toward a Queer Phenomenology*, Ahmed (2006) raises the question: *How do we find ourselves in the places we inhabit?* She argues that the answer to this question depends on the type of body one has, how that body is culturally understood, and the directions in which one is able or permitted to move within a given cultural context. Furthermore, Ahmed maintains that objects, feelings, and opportunities are perceived as closer or more distant depending on one’s physical and discursive starting point. According to Ahmed, *orientation* can occur through various means—some gentle, others harsh—one of which may be the fear or threat of appearing culturally incomprehensible.

This study develops Ahmed’s *queer phenomenology* into *crip phenomenology* (cf. Hall, 2021; Lajoie, 2022) by incorporating dimensions of ableism and disability. Following Reynolds, Hall (2021) describes crip phenomenology as an investigation of disability as lived experience, “not in the form of abstract thought experiments but concretely in a world deeply structured by ableism” (2021, 13). In this article, crip phenomenology offers tools that to examine the becoming of disabled bodies and subjects through the welfare state’s distribution of resources and possibilities. In line with Lajoie (2022), this analysis centers on “the intersection of bodies, worlds, and the everyday practices and norms that determine the intersubjective shape of belonging” (2022, 319).

Applying Ahmed’s understanding, the welfare state can be viewed as *a system of orientation*. By means of its stratification mechanisms, the welfare state redistributes risk from the individual to the collective (Esping-Andersen, 1996; Norberg, 2019; Bylund, 2022). Through bureaucratic tools, economic resources are transformed into services and support, thereby orienting individuals toward specific subject positions. For instance, the Swedish legal reforms governing parental leave enable both women and men to combine parenthood and work life, while support that the LSS Act legislates facilitates disabled citizens’ inclusion in society with self-determination in their daily lives. This orientation is inherently discursive and practical since the welfare state measures and enables individuals to imagine and act on particular possibilities. At the same time, the welfare state has existential dimensions since it shapes who individuals can become. Consequently, the welfare state profoundly influences everyday life’s practical and existential dimensions, from mundane activities like personal hygiene and mobility to access to the labor market and social participation. This includes the embodied and emotional experiences of daily life (cf. Bylund, 2022; Norberg, 2019).

### 2.3 Fear, violence, and combat breathing

In her work on affect, Ahmed defines *fear* as an emotion tied to expectation—we fear that something specific will happen to us. From her perspective, fear is linked to an object, body, or event that approaches us (Ahmed, 2014). Fear is thus culturally constructed and shaped by discourse. In the present analysis, fear emerges from the relationships between the interviewees’ abilities, their dependency on welfare state services, ableist discourse, and austerity measures.

Barbalet (2001) provides a sociological perspective on fear by relating it to a subject’s power in various situations. Drawing on Kemper (1991), Barbalet (2001, 153) argues that fear arises from structural conditions of possessing insufficient power oneself or from the overwhelming power of others. While Barbalet examines fear as a motivator for action in those with power, the present study focuses on his notion of *fear as a response to powerlessness*. Barbalet (2001, 155) also suggests that fear does not always involve a specific threatening agent but can stem from the expectation of adverse outcomes. Similar to the experiences of the Swedish disabled people presented in Norberg (2021), the material analyzed for this study reveals that fear of adverse outcomes—such as a re-assessment of one’s eligibility to receive state support or changes in the state’s welfare eligibility criteria—is central to the precarity experienced by disabled citizens in times of Swedish austerity.

For Norberg, the concept of ‘bureaucratic violence’ is key to understanding how discourse forms systems of biopolitical power through bureaucratic processes in contemporary Swedish austerity measures. In agreement with Nixon (2013, 2), Norberg (2021) argues that “[…]we need to engage a different kind of violence, a violence that is neither spectacular nor instantaneous, but rather incremental and accretive.” Norberg further states (2021, 657) that a distinguishing feature of bureaucratic violence is its “seemingly non-violent nature.” Although the redistribution of resources through the welfare state bureaucracy might appear rational and devoid of emotion, Norberg (2019), Goodley et al. (2018), and McRuer (2018) all show that the impact of austerity measures on disabled people’s lives stems from and produces emotions and affects when enacted. In this article, I employ Ahmed’s concept of ‘affect’ as a productive force to examine the experience of bureaucratic violence in the interviewees’ accounts.

The analysis of the impact of bureaucratic violence on the becoming of a subject is informed by Fanon’s (1970) concept of ‘combat breathing’. Fanon was concerned with state violence in the context of colonialism and argued that ongoing colonial violence reduces the subject to a position where merely staying alive and breathing becomes a struggle (Fanon, 1970, 70). Expanding on his work, Perera and Pugliese (2011, 1) propose that combat breathing is an effect of biopower in various settings where individuals face state violence. This study proposes that austerity constitutes state violence, supported by Perera and Pugliese (2011, 1), who state that there is a “strange intimacy” in violence carried out by the state “at the same time as it is located externally, it shapes the somatic being of the target, amplifying its wounding effects across the body.” The imagery put forward by Perera and Pugliese aptly fits the experiences of disabled people who depend on welfare state support in their everyday lives. As mentioned earlier, for disabled citizens in need of support, changes in the welfare state bureaucracy not only alter the possibilities available for everyday life but also its very experience. Norberg (2021, 667) notes that the stories from disabled people affected by austerity, in her study, are shared by those “that are still alive” opening for the possibility to make a chilling connection between the austerity of the Swedish welfare state and the breathlessness described in Fanon’s concept of ‘combat breathing’ by highlighting the ultimate consequence of austerity politics for disabled people, namely their death. The very act of breathing has also come under scrutiny in the context of Swedish austerity measures targeting services and support for disabled people. A judicial decision once deemed that the assistance provided by managing and monitoring medical breathing devices did not constitute assistance for a “basic need” [as defined in the LSS Act] and, therefore, did not qualify a person eligible for personal assistance. Although this decision was overruled in court in 2019, such (attempted) changes in eligibility bring the issue of breathing to the forefront—not only as a symbol of livability during austerity but also as a stark example of the profound impact that changes to eligibility criteria for welfare state support have on disabled citizens lives (cf. Norberg, 2021, 659).

Bureaucratic changes to the provision of services and support for disabled citizens under austerity measures result in a form of violence that risks being overlooked. The state violence perpetrated through austerity is not, at first glance, as overtly brutal as the state-sanctioned murders that took place in colonial settings that Fanon discussed (1970). However, austerity is predicated on positioning some bodies [i.e., groups of individuals] as subjugated and disposable through specific discourses and economic policies (cf. Ryan, 2019; McRuer, 2018). Perera and Pugliese (2011, 2) draw a connection between Fanon’s concept of ‘combat breathing’ and other types of state violence, arguing that “[o]ne of the key objectives and lived effects of state violence is precisely to reduce the target body to an expendable body who’s right to be is fundamentally questioned…” Thus, the question of expendability lies at the core of austerity measures that target disabled citizens. Austerity measures aimed at reducing or even eliminating services and support for disabled citizens constitute a discursive attack on the personhood of disabled citizens since such measures position them as burdens, parasites, and ‘counterfeit citizens’ (cf. Goodley et al., 2018; Hughes, 2015; Ryan, 2019; McRuer, 2018). Rose et al. (2018) maintain that combat breathing is intimately connected to physiological reactions. They argue that “[c]onsidered as a contested, disfigured daily pulsation, ‘combat breathing’ might be recast as a form of chronic stress,” further citing Herman (2013), who argues that “whereby protracted exposure to ‘a real or perceived threat to homeostasis or well-being[…]can cause pronounced changes in psychology and behavior that have long-term deleterious implications for survival and well-being.” My use of the term *combat breathing* in this study refers to the heightened state of vigilance produced by state violence as manifested in affect. I also consider how this impacts the interviewees, revealing the intimate relationship between austerity as state violence and the becoming of a subjugated subject.

## 3 Method and material

The interview material examined in this study is part of a more extensive set of materials gathered during in-depth qualitative interviews with disabled citizens who required services and support from the Swedish welfare state (see also Bylund, 2022). The interviews were conducted as part of my doctoral research in 2017. Following the interview period, I maintained contact with the participants and made myself available should their living conditions change or should they wish to share additional insights. As a result, the empirical material spans the period from 2017 to 2019. The interview method was grounded in ethnographic and ethnological research paradigms, prioritizing a nuanced, qualitative exploration of individual experiences rather than relying on statistically quantifiable data. The semi-structured interviews, based on open questions, allowed the interviewees to choose what experiences they felt were the most important to share and discuss freely.

The doctoral research project focused on the relationship between welfare state support and the possibilities for disabled citizens in Sweden to engage in romantic relationships, partnerships, and form families. Consequently, a large part of the interview material revolved around changes in the Swedish welfare state, previous experiences, and the interviewees’ hopes and dreams for the future. Fear was a central topic in the interviewees’ accounts of (i) their relationship to the Swedish welfare state, (ii) the process of obtaining state support, and (iii) contemporary austerity politics. For the present study, I have selected the parts of the interview material that focused on accounts of fear caused by austerity.

The interview material was collected under the principle of ‘cross-disability’, which proposes a perspective on disability as a socio-political issue and a heterogenous identity that leads to stigma and marginalization in an ableist society (cf. Bylund, 2022). This principle entailed that the criteria for participation in the interviews were not limited to an individual’s specific medical diagnosis or impairment. By following this principle, I sought to collect a set of a heterogenous materials regarding the interviewees’ disability, gender, age, and class, which made it possible to study the differences and similarities in the interviewees’ experiences based on disability as well as other factors such as gender, socio-economic class and ethnicity. A call for participants was distributed through social media, disability rights organizations, and networks of people involved in disability activism and disability research in Sweden.

A noteworthy aspect of gathering the interview material was my repeated engagement with potential interviewees who expressed ambivalence about participating in the study. They described their relationship with the welfare state bureaucracy as emotionally challenging, and, due to fear that their participation could re-actualize previous traumatic experiences when they claimed state welfare support, they ultimately refrained from participating.

In total, thirteen interviewees participated in the study: four men and nine women. Some were physically disabled, some cognitively disabled or neurodivergent, and some were both physically and cognitively disabled. At the time of the interviews, the interviewees were between 20 and 73 years of age, but most were between 35 and 50 years of age. Many of the interviewees had experience working with Swedish disability rights organizations or were politically active. In this sense, the interview material was relatively homogenous in terms of the interviewees’ prior experience of engaging in matters related to disability rights and applying for services and support from the Swedish welfare state. These shared experiences also influenced their responses and motivation for participating in the study since many of them possessed in-depth knowledge regarding the changes that had taken place in state welfare support for disabled citizens. Their knowledge was based on their work in the disability rights movement, political party involvement, and personal experience.

Most of the interviewees had accessed or continued to access services and support under the LSS Act, including personal assistance, guidance services, accommodation in group homes, or housing with special services. Several interviewees also accessed support provided by the Social Services Act (SoL), such as home help or guidance services. However, many of the interviewees had been impacted by austerity measures from 2009 and onwards and had suffered substantial cuts to their services and support, either at the time of the interview or prior to their interview. Furthermore, some of the interviewees lived entirely without the services and support they needed, having been denied the services they had applied for.

The impact of austerity politics on the practical aspects of disabled citizen’s lives also influenced the choice of research methodology, in response to the inaccessibility and lack of services and support from the welfare state. Following Kerschbaum and Price’s (2016) crip methodology, the interview method focused on providing accessibility for both the researcher and the interviewees. Each interviewee’s ability to perform personal hygiene and everyday tasks such as getting dressed, leaving their home, or traveling determined how the individual interviews were conducted. Most of the interviews were conducted by phone or video calls because many of the interviewees could not travel. In such cases, the interviewees’ needs and their degree of access to state welfare support intersected with my own needs as a researcher. The interview method was thus not only a methodological choice based on accessibility as a principle but also constituted a necessity in times of austerity. As such, this method responded to the doctoral study’s overarching research purpose, i.e., to examine how changes in state support informed the possibilities available to disabled citizens in their everyday lives. The interviewees who received adequate support were often more likely to meet with me in a physical meeting or a video call since their control over their personal hygiene and self-presentation allowed for this. Note that these factors are fundamental to a disabled person’s sense of equality in social interaction. These circumstances also entailed that even if I could meet the potential interviewee in person, the lack of agency in their everyday lives may have led them to refuse participation in an interview. If the possibility of being interviewed by telephone, video call, or chat had not existed, the collected interview material would only have contained stories from individuals who enjoyed enough support and services to meet in person.

The interviews were audio recorded and transcribed. For the sake of their anonymity, the interviewees were given pseudonyms, and the exact details of where they lived were described in general terms, such as “a small town in the south of Sweden” or “in the capital region.” The contents of the transcriptions were initially categorized thematically. These themes were then further analyzed as discourses following a Foucauldian definition of discourse (cf. Foucault, 2010). In the analysis, I classified the interviewees’ accounts as narratives. From an ethnographic point of view, narratives are structured accounts of events and experiences that are shared to convey meaning in specific social, cultural, and political contexts (Langellier and Peterson, 2004). Narratives serve to tell stories and function as a medium through which identities, values, and ideologies are communicated and shaped. From a Foucauldian perspective, narratives are part of discursive formations. They are not just stories but are embedded within power relations and help to reproduce or resist dominant discourses (Foucault and Senellart, 2010; Langellier and Peterson, 2004). Narratives act as tools for organizing meaning while simultaneously shaping how individuals and groups understand their social realities. Based on this theoretical framework, I also paid attention to the ‘silences’ present in the material, made manifest by what the interviewees refrained from talking about and by any contradictions that arose in the interviewees’ different accounts.

### 3.1 Finding fear in the material

Descriptions of fear and anxiety most often emerged in the interviews after I asked the interviewees questions about how they envisioned the future. The interviewees more frequently described various scenarios they were fearful of rather than detailing how they experienced the fear as emotion. Even when they described how past or present fears had affected them physically and emotionally, their responses were often controlled and measured. The interviewees’ seemingly ‘calm’ way in which they described and recounted their experience of strong emotions can be understood through the lens ‘bureaucratic violence’ described above. In systems of welfare state bureaucracy, the ‘non-violent nature’ of bureaucratic violence leads us to communicate calmly about matters vital to our lives (cf. Norberg, 2021). Even interviewees who were in difficult life situations at the time of the interview describe these conditions—and their fear that these conditions will persist or worsen—in relatively calm terms. It was apparent that the interviewees were accustomed to describing their living conditions in contexts where the expression of emotions is not attributed much value, for example, in bureaucratic and legal processes. Furthermore, they had discussed their living conditions on multiple occasions and in various settings before the interview. The interviewees felt and continue to feel fear, but how they described their fear was neither new nor raw. Social anthropologist Tamas (2008), argues that academic work that seeks to bring forth voices about difficult experiences, carries an inherent paradox with respect to depicting trauma and fear:

*We are talking about being broken and undone. But our voices as we speak do not sound broken. [O]ur narrative voice seems to have it all worked out. We know what happened, and we can talk about it in complete sentences that make sense. We can tell others, even strangers, the truth about our experiences. That’s how we turn trauma into knowledge.*

Although Tamas highlights this paradox as a limitation in research into traumatic experiences, I argue that the manner in which the interviewees presented their accounts about austerity politics and bureaucratic violence can be traced back to their experiences with said bureaucracy. For the sake of transparency, I have identified specific elements in their responses that I interpret as expressions of fear when relevant. I highlight these elements in bold typeface and explain how the interviewees framed their experiences.

## 4 Results

### 4.1 The circulation of fear

When they were asked about their thoughts and feelings regarding the future, many of the interviewees referred to the negative experiences of other disabled citizens as examples of what caused them to feel afraid. Several interviewees referred to media segments on the radio or TV which reported on austerity measures that were directed toward people with similar disabilities and living conditions, describing these reports as triggers for their fear.

For example, Ellen, a woman in her late twenties who has cerebral palsy, relied on home-help services from her municipality for tasks such as getting dressed, preparing meals, and household cleaning. Ellen considered that the number of hours of home-help services her municipality had granted her was insufficient for her support needs. However, Ellen was hesitant to apply for more support. When asked about her future, she stated she was worried about keeping the level of support she had at the moment. Regarding this, she referred to what she had heard from others:

*If I had lived with my former partner, now that there is to be a re-assessment, it would probably be much more difficult for me to get support. Then there would have been problems /…/ I am not sure, but I can imagine it. I would probably have received less help. I am not very well-read [on the assessment criteria], but I have heard this from others. Now, it does not affect me very much because I do not have a partner at the moment, but I think it will.* (Ellen)

In Ellen’s account, her understanding of the future was shaped by stories she had come across through her acquaintances in the Swedish disability rights community and in the media. Although examples of strengthening and uplifting narratives exist in the interview material, Ellen predominantly referenced narratives in which disabled citizens had lost their access to services and support from the welfare state. These reports form narratives (cf. Langellier and Peterson, 2004) of potential outcomes for disabled citizens under austerity and function as a form of external monitoring that compels Ellen to reassess her chances of receiving due recognition from the welfare state bureaucracy.

Using these narratives, Ellen creates a scenario that encourages her to orient herself away from specific choices and living conditions that she thinks would jeopardize her eligibility for the services and support she needs. For Ellen, her fear centers around forming a romantic relationship and sharing her home with a partner, something she actively refrains from doing.

The interviewees often spoke of narratives that originated from other places than their own lived experience, such as media coverage of political debates, government propositions, and parliamentary investigations. For instance, between 2016 and 2018, a parliamentary investigation into the existing LSS legislation took place. Initially, the terms of reference for the investigation were informed by an aim that aligned with contemporary austerity politics, i.e., to explicitly reduce costs for personal assistance offered by the Social Insurance Agency and the municipal authorities (Swedish Goverment, 2016). Charlotte, a woman in her seventies at the time of the interview, was one of several interviewees who spoke about the investigation as something that made her quite fearful. Charlotte contracted polio as a child in the 1950’s, forcing her to move to an institution to access education; a life trajectory she shared with many other children affected by polio or other illnesses and impairments at the time. Charlotte was institutionalized in her childhood and young adulthood from the 1950s to the 1970s. After moving out of the institution as a young adult, she lived with home-help services and in residential care until she became eligible for personal assistance under the LSS Act in 1994. When asked what she thought of her future, she responded:

*You never know what will happen with the investigation. They might say that if you are over 65, you will not get any [personal] assistance. We have been there before, and there are many indications that they would present [such a suggestion]. You are never safe when you depend on these services that can change with political decisions.* (Charlotte)

Charlotte’s emphasis regarding how one is never safe when one depends on services and support from the state for one’s everyday life lies at the center of her fear. She reported that she did not feel physically threatened at the moment but remained in a state of heightened vigilance (cf. Perera and Pugliese, 2011). The temporal nature of affect is apparent in Ellen’s and Charlotte’s accounts. When they think of their future, they project a future shaped by austerity measures that negatively affect their everyday lives.

As mentioned, many interviewees had experience working for Swedish disability rights organizations. A personal or professional awareness of current political and bureaucratic processes also seemed to play a part in the feelings that austerity politics evoked. While Ellen described herself as “not being well-read,” Charlotte, who had worked within the field of disability rights for most of her adult life leading up to her retirement, could draw a connection between specific government initiatives (such as the investigation into existing LSS legislation) and a fearful future scenario. The more knowledgable the interviewees were in issues pertaining to disability rights, the stronger their feelings of fear. Like the other interviewees who were in their mid-forties and older, Charlotte had previously lived under conditions radically different from those she lived under at the time of the interview. When she spoke about what she was fearful of (at the time of the interview), she referred to previous experiences. I suggest that fear emerges in a pendulum between temporalities, oscillating between past experiences, contemporary media coverage, political debates, and future scenarios (see Knight and Stewart, 2016). Charlotte’s previous experiences and the detail in which she can imagine her drastically altered living conditions inform the emotional intensity of the negative future she envisions. The affect generated in the present draws on and resonates with past experiences, thereby amplifying her fear.

Several other interviewees share Charlotte’s feeling of “never being safe” since they, too, depend on state support in their everyday lives. Thus, they live in a constant state of *precarity* that previous research has described as a consequence of neoliberal austerity (McRuer, 2018; Saffer et al., 2018). The circulation of affect through the external monitoring of media coverage and personal experiences produces a state of combat breathing through a sense of being encompassed by an ongoing threat where negative consequences that may impact everyday living conditions are a permanent possibility. Under such circumstances, fear is a collective emotion shared by the interviewees; an emotion that does not require physical proximity to a threat (cf. von Scheve and Ismer, 2013). Instead, their positions as ‘disabled citizens’ in contemporary Sweden and their identification with others whom they perceive as their peers enhance their sense of fear. This identification is not primarily based on medical diagnosis or ability, however. In contrast, it is based on the notion of being part of a collective that needs services and support from the welfare state in their everyday life.

When interviewing Jonna, a woman in her mid-forties who lives with progressive muscular atrophy and receives personal assistance from the Social Insurance Agency, this collective identity was brought to the fore. Jonna strongly expressed being affected by and restrained by feelings of fear in her everyday life. However, in contrast with most of the other interviewees, Jonna was content with the number of hours of personal assistance that had been granted to her. Furthermore, she had not experienced any changes in this arrangement for several years. Nevertheless, she still felt that the media coverage of austerity measures and political debates that positioned disabled citizens as an economic burden (cf. Ryan, 2019; Hughes, 2015) impacted her negatively. She reported:

*There is a big difference from, let us say, ten years ago. Then, [personal] assistance was not discussed as it is now. You walk around with a fear of losing what you have. Back then, I thought having a family or living in a relationship was reasonable. Now, it is the case that if I were to move in with a partner, it would lead to me receiving fewer assistance hours and somehow becoming dependent on another person, and I do not want that.* (Jonna)

According to Jonna’s account, her fear revolves around two themes. As in Ellen and Charlotte’s case, she fears losing her state support or having it reduced. Secondly, as a result of that initial fear, she also fears becoming dependent on a person with whom she might enter a romantic relationship. Jonna’s fear of either of these scenarios being realized has led her to live alone, even though she previously wanted a romantic relationship and even start a family. In her life situation, the political and bureaucratic sphere conditions Jonna’s emotional and social orientation.

Like Ellen, fear causes Jonna to orient away from something she previously not only desired but also considered plausible. Following Barbalet (2001), I argue that even though Jonna can be understood as being restrained by her fear, she remains an actor in her life and expresses agency by *not* orienting herself towards the living conditions she desires since she actively avoids seeking out romantic relationships. Narratives from the media and the disability community alike narrow her horizon of possibility. As with positive orientations offered by the welfare state, such as access to personal assistance, the possibility of experiencing more limited living conditions as a result of austerity restricts what she can do in her life and who she can become.

Jonna also indicated how the media image of disabled citizens who need services and support from the welfare state has changed in times of austerity. Under austerity, disabled citizens are viewed as objects of other people’s care rather than citizens entitled to equal living conditions:

*It is more in the general debate now that you are seen as an object that receives care and not an equal person. There has been a shift in values. There have been some strange discussions with my family, too. Like with my sister; I have been worried about how things will turn out, and she has sometimes said: ‘Yes, but if that happens [that you no longer receive personal assistance], then you could move in with me.’ And I think, What are you saying (raising the tone of her vioice)? She wants to tell me that I am not alone; that they are there and will care for me. But I feel even more frightened by that. What if it turns out that way in the end?* (Jonna)

Jonna’s account aligns with previous research on how austerity politics reproduces a discourse in which disabled citizens are understood as ‘undesirable’ in comparison to the neoliberal ideal of a free and productive citizen (McRuer, 2018; Hughes, 2015). When she expresses her fear of how a change in her circumstance could affect her everyday life to her sister, her sister’s response does not alleviate her concerns. Instead, Joanna expresses dismay that her sister has offered to accommodate her in her family home. In Jonna’s example, fear is not only related to proximity but also to probability. Her sister’s kind offer brings the imagined negative scenario even closer to Jonna by confirming that she is not alone in thinking of such a negative scenario. Losing her personal assistance is no longer a secret catastrophic thought that Jonna keeps to herself but is something that others close to her have also contemplated.

Jonna’s account of her conversation with her sister highlights their different ontological, discursive, and epistemological positions. Although Jonna and her sister share a close relationship, their lives are dramatically divergent in a society shaped by ableism. This divergence is due to differences in their abilities, bodies, and need for support and services in their everyday lives. The impact of austerity politics that Jonna experiences is not experienced by her sister, even though her sister empathizes with the obstacles that austerity policies create in Jonna’s life. For Jonna, her fear of potential negative consequences and their outcomes induces a sense of disorientation. As Lajoie (2022) has shown, disorientation occurs when “habits, gestures, or patterns of thought are called into question” (2022, 331). While such experiences may happen to everyone during the course of their life, Lajoie (2022) argues that for most, such experiences do not undermine their fundamental sense of belonging in the world. However, for disabled subjects, disorientation is often more profound, long-lasting, and structurally imposed, frequently involving physical, cognitive, or bureaucratic barriers. According to Lajoie (2022), this means that the disorientation experienced by disabled people compromises their sense of belonging in the world. In the case of Jonna and her sister, their respective subject positions not only create different living conditions but also shape their perceptions of what is ‘dangerous’ or ‘safe’. For Jonna, being cared for by her sister does not foster a sense of safety but, instead, evokes a feeling of dread.

Jonna’s, Ellen’s, and Charlotte’s accounts of what they fear reveal what they perceive as the most significant threat of austerity politics: living with a lack of self-determination and being dependent on others. Butler (2009) discusses how specific lives, bodies, and subjects are constructed as ‘grievable’ depending on how they relate to the hegemonic discourse in the surrounding culture. Butler (2009) argues that grievable lives are recognizable to the majority of people and are understood as ‘worthy of protection’. McRuer (2018) has further developed the concept of ‘grieveability’ in neoliberal austerity policies so as to include lives or subjects who are understood as productive or profitable, which is in line with the thesis of ableism. In discussing what they fear, the interviewees relate to notions of ‘liveability’ rather than ‘grieveability’. The traded narratives underscore the circulation of affect and the idea that collective emotions, which are rooted in a sense of belonging to a specific social group, do not necessarily require physical proximity to a threat. The sense of sameness, with regard to their life circumstances, that enables the interviewees to relate to the narratives of others is informed by a combination of personal experiences, physical or cognitive abilities, and a shared need for services and support from the welfare state. This sameness of experience, in turn, creates a socio-political position that emerges when changes are made to the bureaucratic governance and distribution of welfare state services. Narratives of adverse experiences of others, such as those reported in the media, heighten the interviewees’ awareness of these issues, making them fearful of facing similar negative consequences in their own lives.

### 4.2 Letters, phone calls, and e-mails: objects of fear

Many of the interviewees described how their fear was directly linked to previous experiences of their welfare state support being (re)assessed. Ellen stated that:

*Applying for support is always tricky because you are constantly questioned. Every time there is a re-assessment, you are terrified that the support you have will be withdrawn /…/ because they have their rules: ‘We can grant you this, but we cannot grant you that.’* (Ellen)

In Ellen’s account, her fear appears as a structural condition of insufficient power, as noted by Kemper [in Barbalet (2001)]. When the interviewees apply for services and support during a time of austerity, they enter into an asymmetric power dynamic. Norberg (2021) has contextualized this dynamic as ‘bureaucratic violence’ that is made manifest physically in meetings between the person applying for support and a Social Insurance Agency or municipal case worker. Norberg argues that “(re)assessments are also contexts where disabled people have little power if they feel that the assessment is inappropriate” (2021, 662). In the interviews recorded for this study, expressions of being “made to,” “forced,” or “not having a choice” are prominent in the interviewees’ accounts of the assessment and re-assessment procedures they have been subject to.

For instance, Marcus, a forty-year-old man with cerebral palsy, who lived with his wife and two daughters at the time of the interview, described how he felt increasingly worried the nearer he was to a re-assessment session regarding his personal assistance at his municipality. Since he had lost his right to personal assistance from the Social Insurance Agency in 2013, the municipal re-assessments had become increasingly frequent. Sometimes, they were only 6 months apart. He provided the following account:

*I was very anxious that an envelope with a review decision would arrive in my mailbox. I waited every day for it between 2012 and 2013. Your pulse rises when you see a letter with the Social Insurance Agency’s or the municipality’s logo. It is a real threat, an external threat, to your whole life.* (Marcus)

The physical symptoms of fear presented by Marcus were shared among the other interviewees. Marcus, Charlotte, Eva, Agnes, and Ida described how a general fear of austerity measures gradually transformed into physical reactions and avoidant patterns in their everyday lives. In each of their accounts, they provide several examples of feeling terrified if a municipal case worker calls them on the telephone or if they receive an e-mail from the Social Insurance Agency. Jonna mentioned that, at times, she actively avoids collecting her mail because she is too afraid of seeing a letter from the Social Insurance Agency. Such a letter would cause her anxiety levels to ‘skyrocket’, she added.

In these accounts affect is simultaneously located both inside and outside the body. Letters and phone calls become imbued with what Butler calls “accumulated violence” (Butler, 1997, 52), which reactivates previous experiences of bureaucratic violence associated with assessments or re-assessments. Such experiences evoke a lack of control over the future and a morbid anticipation of its potential adverse outcomes. When charged with accumulated violence, these objects transform the interviewees’ bodily experience and induce a state of combat breathing and a heightened vigilance that is accompanied by headaches, anxiety, and heart palpitations. For individuals who do not rely on services and support from the welfare state but have a disability, a call from the municipality or a letter from the Social Insurance Agency may signal that these authorities are ready to provide assistance or help. However, in the case of the interviewees included in this study, the austerity measures that were in place at the time of the interview had shifted their relationship with these forms of communication from a sense of security to one of dread and perceived threat.

In Marcus’ case, for example, any contact with the Social Insurance Agency or the municipality actualized his past experience in performing an ADL (Activities in Daily Life) assessment to confirm his support needs. During said assessment, a vocational therapist was asked to observe Marcus in real-time while he was being assisted in taking a shower. “I had to,” Marcus stated during his interview. “I could not risk, for the sake of my children, not being given any support.” In Marcus’ case, bureaucratic violence (Norberg, 2021) not only breached the verbal boundaries of personal integrity, but even physical and practical acts that targeted the most private parts of everyday life. For Marcus, the fear he experienced, and his physical reaction are not abstract and merely driven by media narratives of a perspective in a political debate. Marcus’ combat breathing sprang forth from the very real and physical experience of having to submit to a violation of his personal integrity. In this instance, the use of Fanon’s concept of ‘combat breathing’ highlights the close relationship between restrictive eligibility criteria for services and support during austerity, increased control over the recipient of said services and support through bureaucratic tools, and genuine physical and mental harm.

### 4.3 Wounding affects: consequences for one’s mental and physical health

Some of the interviewees spoke about profound physical reactions or long-term impacts of living with the consequences of austerity politics. Mia, a blind woman in her mid-forties, had had drastic changes made to her services and support conditions. At the time of the interview, she had a home-help permit from her municipality to help her with cleaning around the house and shopping. However, she lacked guidance services that would enable her to participate in social events, leisure activities, and physical exercise. Mia described the physical effects of the lack of support in the following:

*I became depressed and gained a lot of weight because I was only at home and comforting myself with food. /…/ I felt like my whole life was a bureaucratic obstacle. I had to start taking antidepressants to cope. It is a constant stress when you do not know how life will turn out or what the next assessment will bring. It is not possible to plan your life. I will always have a visual impairment, but I hope that I will not always have my depression.*

Mia described how the lack of support and services causes her to worry about her future and has an impact on her self-image, thus her depression. A lack of physical activity in her everyday life combined with depression prompts her to turn to food for comfort, further impacting her health and sense of self negatively.

Ida, a woman in her forties with cerebral palsy, was also the recipient of home-help services from her municipality. She and her husband, who also had a physical disability, had been through numerous assessment and appeal processes so as to get enough support to take care of their child. She reported how these processes affected her husband’s mental health:

*My husband could not take the pressure in the end, and then it was as if they woke up at the municipality. So, you could say that for us, it took a trip to the psychiatry ward for them to realize what was at stake.* (Ida)

Ida described how the existential threat, to their family life, and the stress of the bureaucratic process resulted in specific psychological consequences for her husband. This, in turn, apparently prompted the municipality case workers to “wake up.” For Ida, it was only when the stress took on measurable consequences in a medical sense, with a diagnosis, that the case workers seemed to consider the importance of the support and services that she and her husband needed. Ida’s experience aligns with observations made in previous research on how a medical discourse becomes increasingly hegemonic in defining the specific needs or living conditions that render a disabled person eligible for services and support from the welfare state. Previous research has demonstrated that a discursive and legal shift has changed the aims of the support that is provided. The aims have changed from support being a tool for social inclusion and satisfaction of civil rights to a medicalized approach to providing support where only needs considered *integritetsnära* (‘pertaining to one’s personal integrity’) render one eligible for support. Following this, a fragmented approach to assessing the needs of the disabled individual, where, for example, needing help with getting dressed in a coat and shoes, does not count as ‘support with getting dressed’ (cf. Berggren et al., 2021; Lewin, 2021). In this context, the fact that Ida’s husband was visibly affected by the process that he had to follow so as to obtain services and support plays a crucial part in Idas’s understanding of what it actually was that made them eligible for the support they needed, i.e., a measurable condition in a medical discourse.

Agnes described a similar situation. She was one of the youngest interviewees, a woman in her twenties living with multiple physical disabilities. At the time of the interview in 2017, Agnes had been involved in an appeal process for the right to personal assistance from her municipality for several years and continued to be so during the following years. In 2019, she sent me a message saying she had been hospitalized for several weeks due to problems with her breathing and blood pressure. It was ultimately concluded that she had developed a chronic illness affecting her lungs and that she would need daily medication and breathing aids. When Agnes’s doctor learned that she had been under immense emotional pressure throughout her appeal processes and that a lack of services and support had prevented her from taking proactive action regarding her deteriorating health, he attributed her newly diagnosed medical condition as being caused by a lack of services and support. As in Ida’s case, Agnes also hoped these measurable and documented physical consequences of lack of adequate support and services would lead to positive change in her everyday life. “Maybe someone can understand the seriousness of the situation now,” she remarked.

Ida’s and Agnes’ accounts reveal the double-edged sword of medical bureaucratization with regard to disabled citizens’ bodies, lives, and possibilities. This issue is further discussed by Lajoie (2022) in the case of accessibility. In Ida’s and Agnes’ cases, the negative consequences of bureaucratic violence and a lack of support may increase their eligibility for state support since medically measurable negative consequences underpin their needs.

Another interviewee, Leon, a trans man in his late thirties, described how the process of applying for support and making his needs and illness comprehensible to a bureaucratic system also had an impact on his well-being. At the time of the interview, Leon underwent a set of medical investigations that ultimately diagnosed him with Myalgic encephalomyelitis (ME), a chronic illness. One of the symptoms of ME is Post Exertional Malaise (PEM), which may cause a permanent deterioration in the patient’s physical health. PEM can be triggered by everyday tasks such as showering, cooking, or taking a walk. However, emotions such as fear, stress, or anticipation of a negative event can also trigger PEM for those most severely affected by the illness [National Institute for Health and Care Excellence (NICE), 2021]. Leon reported how his fear of the consequences of austerity politics, combined with inadequate services and support, caused his symptoms to worsen:

*I get sick from all the doctor’s visits and the workload that bureaucracy entails. If I could get away from that and not be questioned and scrutinized all the time, I would feel better.* (Leon)

In this section, I have presented the interviewees report on how prolonged stress and an emotional state of fear and anxiety, in conjunction with the practical obstacles caused by austerity, have mental and physical consequences. I interpret their accounts as examples of being in a state of combat breathing and its mental and physical effects. Since the 1990s, medical studies have documented that the strain of discrimination leads to poor mental and physical health (Guidi et al., 2014). For example, physical illness due to material and social marginalization is described as an *allostatic load* (ibid.). This term describes the mental and physical strain that an individual experiences if their body’s stress reactions are frequently activated or activated for a prolonged period. If the perceived threat that produces a stress reaction is not averted or mitigated, the body is put under constant mental and physical tension that leads to a (measurable) physical illness. Hence, Frantz Fanon’s ‘combat breathing’ concept is an apt metaphor for the consequences of enduring state violence. Paired with the findings of medical research it can be said to describe an actual physiological process that is associated with measurable, physical and psychological consequences.

### 4.4 “It cannot happen here”: disorienting affects

Marcus disclosed that the precarious situation he faced—marked by inadequate and short permits for services and support often re-assed every six or twelve months—had, at times, caused his anxiety levels to rise so high that he had been unable to function in his daily life. However, when he sought help from a psychologist to manage his anxiety, the psychologist found it challenging to make sense of his situation. Marcus considered why this was the case:

*In Sweden, we do not believe that the state can treat a citizen like this, that it just keeps on happening. There is no language to explain what is happening. /…/ If this had been a relationship, I would have ended it, but how can you leave your municipality?* (Marcus)

Marcus’ poignant account describes how being the subject of constant re-assessment left him feeling being stuck in a destructive or dysfunctional relationship where traumatic events are repeated. As a disabled person in need of services and support in his everyday life, he foresees that he will always be in some form of relationship with a municipality or the Social Insurance Agency. In Marcus’ analogy about being trapped in a destructive relationship, a discursive silence emerges around experiences of the Swedish welfare state as ‘violent’ (cf. Norberg, 2021). Foucault (2010) emphasizes that discourse not only constitutes knowledge but also regulates what *can* be known or said within a particular cultural or historical moment. In Marcus’ case, the hegemonic discourse of a ‘just and fair’ Swedish welfare state is challenged. Marcus felt that his position was as difficult to articulate as the trauma that had initially caused him to be in that position in the first place. The hegemonic discourse of the Swedish welfare state as ‘fair and just’ offers no language to describe the violence it perpetrates. Accounts of struggling to make sense of their situation when they meet with abled-bodied friends, family members, professionals, or colleagues were reported by several other interviewees. They declared that they could not align themselves with the hegemonic discourse of citizenship and the ideal of a ‘just and fair’ Swedish welfare state. Instead, this lack of alignment causes them to experience a sense of disorientation. Lajoie (2022) has explored how ableist lifeworlds disorient disabled people and “seriously impede the experience of belonging” (332). When the disabled citizens in this study attempt to articulate their physical and emotional experiences of bureaucratic violence to others, they find that the hegemonic narratives surrounding citizenship clash starkly with the actual conditions of their lives.

Previous research on emotions and citizenship has shown that citizenship, aside from being a legal definition of a person’s status in a nation, is constructed by and produces emotions centered around the concept of ‘belonging’ (Ho, 2009; Fortier, 2016). In line with previous research on ‘affective citizenship’ (Fortier, 2016), I argue that the effects of austerity politics radically alter the experience of citizenship and the feeling of belonging. If citizenship is a question of belonging, ableist austerity centers around separating out individuals who are categorized as ‘not contributing enough to belong’, i.e., their right to belong is somehow annulled by their perceived inability to contribute to society. Furthermore, the hegemonic discourse of the well-functioning Swedish welfare state is also based on the notion of a citizen being protected and supported. The interviewees’ experiences have tarnished this hegemonic concept of Swedish citizenship, leaving them feeling violated, coerced, and fearful, further impeding their sense of belonging. The emotions generated by these experiences erode their trust in the state’s ability to safeguard their rights and provide adequate services and support services. Consequently, their sense of disorientation extends beyond personal aspirations and desires, revealing how citizenship and rights in the welfare state are, in fact, unevenly distributed on account of a person’s disability. The disorientation produced by bureaucratic violence exposes an ableist hierarchy that is embedded in austerity measures and is thus also latent in the welfare state’s redistribution of resources, where certain citizens are deemed worthy of being safe while others are not (cf. McRuer, 2018; Ryan, 2019).

## 5 Conclusion

In the accounts analyzed in this study, fear emerges as a distinct part of a collective emotional landscape of Swedish austerity politics aimed at reducing the services and support for disabled citizens between 2009 and 2019. Fear circulates in the form of narratives traded between disabled citizens and in the form of personal experience, media coverage, and political debate. Fear alters the meaning of everyday actions and objects, for example, answering the phone or collecting the mail. Fear also constitutes a wounded body, inflicting harm both physically and mentally as it produces heightened levels of stress and anxiety. Fear has disoriented the interviewees away from their everyday dreams and desires. Bureaucratic violence governed by austerity is the basis of the production and circulation of fear. As such, fear should be viewed as a symptom of disabled citizens’ marginalization under conditions of neo-liberal austerity (Ryan, 2019; McRuer, 2018).

Seen through the lens of Fanon’s concept of ‘combat breathing’, the findings of this study add to previous research that has argued that disabled citizens exist in a heightened state of emotional vigilance in times of austerity (Hughes, 2015; Norberg, 2021). The concept of ‘combat breathing’ does not necessarily signify that one is prepared or able to fight back. Instead, *combat breathing* can refer to a heightened state of vigilance as a consequence of an external threat (Perera and Pugliese, 2011). The effects of fear constitute a driving force in this mechanism of heightened vigilance. This study’s findings also reveal how affects are made manifest in the body in a manner that strongly suggests that *bureaucratic violence has consequences equivalent to direct, physical violence*.

In response to Goodley et al.’s (2018) call for the use of affect theory in disability studies as a tool to further investigate the consequences of ableism, the findings of this study reveal an intricate relationship between welfare state governance and the emotional lives of disabled citizens. Consequently, examining how emotion and affect are circulated between political governance, societal discourse, and individuals can provide valuable insight into the importance of emotion in the production of disabled citizens’ sense of self and sense of safety.

Fear as a characteristic affect for disabled citizens in times of austerity stems from, (re)produces, and impacts how the interviewees experience physical and mental states. Furthermore, fear determines their orientation toward and away from various actions. As such, fear as affect “define[s] and ceaselessly constitute[s] and reconstitute[s] the nature of a body” (Seyfert, 2012, 37). However, since the production of fear can be traced to specific political, legal, and bureaucratic changes in the welfare state’s provision of services and support, it prompts us to ask the question: Would a different discourse and governance create a different affective landscape? As previously mentioned, the interviewees described different scenarios that they are fearful of more often than they described how the fear felt. This way of presenting their feelings, in terms of possible scenarios or previous experiences, reveals the profound connection between the interviewees’ dependence on welfare state support and the production of affects. When asked to describe their feelings, the interviewees could not detach their feelings from the bureaucratic and political landscape that formed their everyday life.

The fear that is produced by austerity measures limited the interviewees’ ability to imagine and act towards securing a prosperous future, even if they were not physically or practically limited in doing so at the time of their interview. This observation aligns with Saffer et al.’s (2018) argument that fear produces a ‘limited subject’ who self-restricts out of fear of further restrictions. In this regard, fear as an affect is a symptom of previously experienced trauma and is a traumatic infliction on its own. When one is in a state of fear caused by austerity politics, mundane tasks such as collecting the mail, reading a news report, or engaging in a conversation with friends and family can result in a state of combat breathing. This study’s findings also support what Watermeyer and Swartz (2016) has described as ‘a battle on two fronts’. Disabled citizen’s not only experience material and economic marginalization and a lack of services and support, they also face emotional and existential violence caused by the fear of political, legal, or bureaucratic measures that will enhance this marginalization. Under such circumstances, fear is inherently disorienting because it prevented the interviewees from engaging in things they want or desire for fear of suffering adverse consequences if they did so. The path before them may be open, but they dare not travel along it. However, following Barbalet (2001), I categorize ‘choosing not to act on wants and desires’ as a deliberate action, not merely a state of inaction or paralysis.

Disorientation (as discussed above) is also related to the notion of ‘Swedish exceptionalism’, where the welfare state is presented as an inherently just system of stratification that keeps citizens safe (Norberg, 2019). If the welfare state produces adverse affects such as fear, these affects not only cause physical and emotional harm; they also disorient the subject from family members, healthcare professionals, and other citizens. This study thus also contributes to the field of affective citizenship by revealing how the experience of citizenship is not only a question of nationality and belonging but also a question of dis/ability and the biopolitics of the welfare state.

In conclusion, analyzing the feelings of disabled citizens provides valuable insight into the existential and physical experiences of ableism while also revealing the discursive landscape and governance of the surrounding society—an area that warrants further research.

## Data Availability

The raw data supporting the conclusions of this article will be made available by the authors, without undue reservation.
